# In memorium, Michael Zigmond, PhD

**DOI:** 10.1038/s41531-023-00597-8

**Published:** 2023-11-01

**Authors:** Richard J. Smeyne

**Affiliations:** Department of Neurosciences, Thomas Jefferson University, Philadelphia, PA USA

**Keywords:** Scientific community, Neuroscience

Dr. Michael Zigmond (b. 9/1/1941) died on 28 Aug 2023 at age 81 from complications of Amyotrophic Lateral Sclerosis (ALS, Aka Lou Gherig’s Disease). His work had a great impact on the world. He was an educator, a leader, and an inspiration and role model to an enormous number of people. He was a person of great integrity and compassion. He was driven to do good. He was immensely kind. He dwelled on possibility. He was not constrained by what things were like now, or by what was practical. He dreamed. He dreamed big. But he brought his dreams to fruition. He put ideas into action, and he made them happen. That is just one of the ways Michael cared for other people and other people’s welfare. And he truly did care for all people. He made friends with everyone. Everyone. In addition to the vast number of colleagues, he also talked to the janitors, he talked to the maids, he talked to the homeless people. Michael was always innovating, trying to make things better. You could see the gears in his brain going, he would latch onto ideas and run with them. He did not think about what he could do, he thought about what WE could do. What all of us could do. Some scientists do not share their ideas. They have lab meetings with closed doors. Anytime data or ideas are discussed, they close it to their group. But not Michael. He knew that great things were built by collaborations with others. He was such an incredible networker. There was actually a joke among members of his lab that that he could get onto an elevator at the top of a building and by the time he came out at the bottom floor he would have made a new collaboration. It was this aspect of his personality that he built an incredible research team, with collaborators in the offices nearby his, and as far away as India, China, and South Africa.

Michael was a pioneer in some of the early studies on Parkinson’s disease. He was one of the first scientists to recognize the beneficial effects of exercise in slowing the progression of this disease; studies opened up an entirely new avenue of research that has produced one of the best clinical treatments for slowing Parkinson’s disease progression. In his later career, his work focused on work examining the interface of brain health and disease, with a particular focus on the impact of social isolation including that seen in solitary confinement. His research on the long-term effects of solitary confinement has contributed to numerous states’ legislatures changing their regulations in regard to this practice.

Dr. Zigmond received his B.S. from Carnegie Institute in Chemical Engineering in 1963 and then attended the University of Chicago where he earned his PhD in Biopsychology in 1967. Following his PhD, Dr. Zigmond moved to MIT where he did postdoctoral studies in the lab of Richard Wurtman. In 1970 he joined the faculty at the University of Pittsburgh, first in the Faculty of Arts and Sciences (1970–1988) and then in the Department of Neurology (1988–2018). In 2019, he was honored as Professor Emeritus. While on the faculty at the University of Pittsburgh, Michael, along with Bob Moore were instrumental in the formation of the Pittsburgh Institute for Neurodegenerative Disease; which today is thriving and making important contributions to the development of new therapies for a number of neurological diseases. In addition to being a founder of the PIND, in 2004 Michael also was PI on a prestigious award from the NIH, where his laboratory and others were awarded a Udall Center of Excellence by the National Institute of Neurological Disorders and Stroke (NINDS).

While at the University of Pittsburgh, Michael ran a research program sponsored by NIH and DOD that was focused on brain neurobiology in health and disease. Using cellular and animal models of Parkinson’s disease, Dr. Zigmond has explored the relationships between stress, exercise, and biochemical factors on the survival of dopamine neurons, which are depleted in the neurodegenerative disorder. In terms of his research, Michael was one of the earliest bench scientists to focus on developing animal models of Parkinson’s disease. For example, he along with his colleague Ed Stricker and other collaborators were some of the first to develop the 6-OHDA dopamine depletion animal model, which was the standard for animal studies prior to the discovery of MPTP in the 1980’s. While at Pittsburgh, Dr. Zigmond’s lab focused on three important questions related to Parkinson’s disease. First, what are the neurobiological deficits that often occur during aging, and can any of these aging effects be altered by interventions such as trophic factors and/or exercise? Second, his lab asked what underlies the loss of dopamine neurons in Parkinson’s disease. These studies examined the signaling cascades (e.g., Ras/Erk, PI3K/Akt) and their role in neuronal resiliency. A third area of study examined how stress reduces or increases (preconditioning) the vulnerability of the brain to subsequent insults. In the later stages of his research career, Dr. Zigmond also focused on examining how social isolation affected the brain. In addition to his own studies, Dr. Zigmond had an extensive network of collaborators. Michael is also a dedicated mentor. During his career, he mentored some 50 graduate students and postdoctoral fellows, more than half of whom were women. He also served as a mentor to a number of junior faculty. This extensive network of scientists, many of whom are still active in the field, will continue Michael’s scientific legacy.

During his career, Dr. Zigmond published close to 175 original research papers, 30 reviews, and numerous book chapters. He was senior editor of the books Fundamental Neuroscience (1999) and Neurobiology of Neurological and Psychiatric Disorders (2022) and served as Editor-in-Chief for *Progress in Neurobiology* from 2000 to 2018. He had a life-long H-Index of 86 and was listed in the top 500 Neuroscientists (all time) in the USA by research.com.

He served on numerous committees, including those of the National Academy of Sciences and other important organizations, to develop guidelines and recommendations to provide explicit training in professional development and research ethics as part of a research training program. He was very gratified to see that it’s now a requirement on federal training grants, that one must provide these sorts of skills, and that a plan of mentoring must be included for fellows.

Michael was described by his colleagues as having the greatest integrity ever seen in a human being. In fact, it was this “integrity” that led to the development of a series of ethics workshops, including those dealing with the responsible conduct of research. Along with his long-time collaborator, Dr. Beth Fischer, these workshops focused on professional skills and responsible conduct of research; and evolved to a program called “The Survival Skills and Ethics Program.” and have been subsequently taught internationally including in the United States, Europe, Africa, Asia, and the Middle East. Importantly, the skills taught in these classes were designed for students, and junior and senior faculty in a “train-the-trainer format” so that the critical information presented could continue to have an impact beyond the direct teaching of the course. It is important to note that these workshops were instituted well before cases of scientific misconduct started to make headlines in newspapers. He felt that it was important to teach the ethical dimensions of these skills alongside all of the other technicalities. And not to bring in people from the humanities or some other departments that might know ethics but did not understand the practice of science.

Over his distinguished career, Dr. Zigmond received numerous awards and honors. In 2009, he was elected as a Fellow of the American Association for the Advancement of Science. In 2012, Michael received the Mika Salpeter Lifetime Achievement Award at the annual meeting of the Society for Neuroscience in New Orleans. According to the Society, “the award recognizes individuals with outstanding career achievements who have also actively promoted the professional advancement of women in neuroscience.” In addition to individual honors, Michael also served as an advisor to the National Academy of Science (NAS) in the production of several of their manuals, including “On Being a Scientist; Advisor, Teacher, Role Model, Friend*”*, and “Enhancing the Postdoctoral Experience*”*. He was also a member of a U.S. Institute of Medicine task force on research integrity that produced “Integrity in Scientific Research.*”* He served as President of the Association of Neuroscience Departments and Programs in 1991 and received its Award in Education in 1999. In the Society for Neuroscience, Michael held numerous leadership positions. These include his election as Secretary of the SFN (1994–1996). He also served on the Minority Education, Training, and Professional Advancement Committee (1997–2000), the Social Issues Roundtable Committee (1996–2002), and the Committee on Development of Women’s Careers in Neuroscience or CDWCN (1999–2003). He also chaired the committee that wrote the first SFN’s “Guidelines for Responsible Conduct Regarding Scientific Communication*”*.

He has organized a number of programs to promote diversity, made presentations at conferences of underserved individuals, chaired the Program Advisory Committee for the NIH-sponsored program at Universidad Central del Caribe in Puerto Rico, and served on an advisory committee at the University of Puerto Rico. In the later years of his work, Michael dedicated his time and efforts to working to eliminate the use of long-term solitary confinement in the criminal justice system. Working with legal scholars, neuroscientists, psychologists, advocacy groups, state and local legislatures as well as persons in the criminal justice system (both those incarcerated as well as those in its administration), he vigorously spoke out and worked to change what he called “this inhumane” form of punishment. Michael’s advocacy has resulted in changes in a number of state’s criminal codes and his studies, including one paper on the neurobiological changes induced by prolonged isolation, published 2 weeks prior to his death, will continue to have a significant impact on this topic.

One final note. Following his retirement from the University of Pittsburgh, he moved to Woods Hole, MA. Like his earlier life in Pittsburgh, Michael became a leader in this community. He was a peacemaker and fought for equity, diversity, and inclusion. This dates back to the time in graduate school. At that time, he was a self-described pacifist who resisted the Vietnam war, despite the imminent threat that he would be jailed for it. He ran tours to promote peace in the Middle East. And he worked for diversity and social justice. He did not shy away from those in the shadows. He made friends with everyone. One of his last efforts was to try to address the issue of homelessness on Cape Cod, which remains today a very real problem. Michael often quoted Rabbi Abraham Joshua Heschel who said “indifference to evil is worse than evil itself, that in a free society, some are guilty, but all are responsible.” And I think he truly believed that to his core.

Michael is survived by his wife Naomi, two children (Daniel and Leah), and 4 grandchildren. He is also survived by his brother, Richard, currently a Professor in the Departments of Neuroscience, Neurological Surgery, and Pathology at Case Western School of Medicine in Cleveland, OH. May his memory be a blessing.

